# Hemispheric Differences in White Matter Microstructure between Two Profiles of Children with High Intelligence Quotient vs. Controls: A Tract-Based Spatial Statistics Study

**DOI:** 10.3389/fnins.2017.00173

**Published:** 2017-04-03

**Authors:** Fanny Nusbaum, Salem Hannoun, Gabriel Kocevar, Claudio Stamile, Pierre Fourneret, Olivier Revol, Dominique Sappey-Marinier

**Affiliations:** ^1^Laboratoire Parcours Santé Systémique (EA4129), Université Claude Bernard-Lyon 1 & Centre PSYRENELyon, France; ^2^CREATIS (CNRS UMR5220 & INSERM U1206), Université Claude Bernard-Lyon 1Villeurbanne, France; ^3^Faculty of Medicine, Abu-Haidar Neuroscience Institute, American University of BeirutBeirut, Lebanon; ^4^Service de Psychopathologie du Développement, Hôpital Femme-Mère-Enfant, Hospices Civils de LyonBron, France; ^5^Service de Psychopathologie de l'Enfant et de l'Adolescent, Hôpital Neurologique, Hospices Civils de LyonBron, France; ^6^CERMEP—Imagerie du Vivant, Université de LyonBron, France

**Keywords:** high intelligence-quotient children, gifted, talented, MRI, diffusion tensor imaging, tract-based spatial statistics (TBSS)

## Abstract

**Objectives:** The main goal of this study was to investigate and compare the neural substrate of two children's profiles of high intelligence quotient (HIQ).

**Methods:** Two groups of HIQ children were included with either a homogeneous (Hom-HIQ: *n* = 20) or a heterogeneous IQ profile (Het-HIQ: *n* = 24) as defined by a significant difference between verbal comprehension index and perceptual reasoning index. Diffusion tensor imaging was used to assess white matter (WM) microstructure while tract-based spatial statistics (TBSS) analysis was performed to detect and localize WM regional differences in fractional anisotropy (FA), mean diffusivity, axial (AD), and radial diffusivities. Quantitative measurements were performed on 48 regions and 21 fiber-bundles of WM.

**Results:** Hom-HIQ children presented higher FA than Het-HIQ children in widespread WM regions including central structures, and associative intra-hemispheric WM fasciculi. AD was also greater in numerous WM regions of Total-HIQ, Hom-HIQ, and Het-HIQ groups when compared to the Control group. Hom-HIQ and Het-HIQ groups also differed by their hemispheric lateralization in AD differences compared to Controls. Het-HIQ and Hom-HIQ groups showed a lateralization ratio (left/right) of 1.38 and 0.78, respectively.

**Conclusions:** These findings suggest that both inter- and intra-hemispheric WM integrity are enhanced in HIQ children and that neural substrate differs between Hom-HIQ and Het-HIQ. The left hemispheric lateralization of Het-HIQ children is concordant with their higher verbal index while the relative right hemispheric lateralization of Hom-HIQ children is concordant with their global brain processing and adaptation capacities as evidenced by their homogeneous IQ.

## Introduction

Children with very high intelligence quotient (HIQ) [superior to 130 as measured by the fourth version of the Wechsler Intelligence Scale for Children (WISC-IV) (Wechsler, 2005)] are referred to as “gifted,” “talented,” as well as “high potential.” However, these terms imply different characteristics and conceptions that remain misunderstood. Qualitatively, HIQ children are perceived as having all or part of the following characteristics: faster processing speed based on a rich vocabulary range, greater attention, and visuo-spatial abilities, fast and large memory, superior problem solving capacities, mental flexibility, and reasoning strategies (Vaivre-Douret, 2011). Nonetheless, these exceptional characteristics are not always associated with learning or educational achievements. This paradox was investigated during the last decades but remains misunderstood (Barchmann and Kinze, 1990; Reis and McCoach, 2000).

Although gifted in certain areas of the curriculum, some HIQ children may present different disabilities in reading, writing, coordination, attention, and/or managing their emotions and relationships. Indeed, many HIQ children cases are referred to pediatric or child neuropsychiatry departments for socio-emotional problems such as anxiety, social withdrawal, low self-esteem, excessive perfectionism (Guignard et al., 2012), deficit of attention and/or school underachievement (Guénolé et al., 2015). In contrast, other HIQ children, corresponding better to the “talented” or “gifted” appellation, seem to be well adapted to their environment. These clinical observations have led us to define two profiles of HIQ children. On one hand, the homogeneous HIQ profile (Hom-HIQ) usually presents quite well-controlled behavior and successful curriculum. On the other hand, the heterogeneous HIQ profile (Het-HIQ) can show emotional and social maladjustment as well as learning troubles that can be related to a “dyssynchrony syndrome” (Terrassier, 2009; Guénolé et al., 2015). Such heterogeneity, also called “developmental asynchrony” by Alsop (2003) and Silverman (2011), can be detected by the Wechsler's IQ test. Indeed, a significant difference between verbal comprehension index (VCI) and perceptual reasoning index (PRI) values of WISC-IV as well as standard levels in working memory index (WMI) and processing speed index (PSI) are considered as significant markers indicative of a heterogeneous profile (Berk, 1982; Bessou et al., 2005; Sweetland et al., 2006; Guénolé et al., 2013).

Diffusion tensor imaging (DTI) is a unique non-invasive technique that provides microstructural information related to white matter (WM) integrity (Basser et al., 1994; Basser and Jones, 2002) and allows the identification of neural pathways (Beaulieu, 2002). In addition to various clinical applications, DTI has been used to study normal development of WM that is thought to play a key role in cognitive functions (Qiu et al., 2008; Lenroot and Giedd, 2010; Schmithorst and Yuan, 2010). Indeed, as distant brain regions become more efficiently interconnected, it is expected that the ability to transfer and analyze information also becomes more efficient. Thus, the high sensitivity of DTI to detect WM changes has led to recent investigations of neural connections in children and adolescents with specific abilities such as arithmetic (Tsang et al., 2009; Van Beek et al., 2014), executive functions (Seghete et al., 2013) as well as general intelligence (Schmithorst et al., 2005; Gläscher et al., 2010; Barbey et al., 2013; Dunst et al., 2014). Particularly, Clayden et al. (2012) demonstrated that increased anisotropy in the splenium of the corpus callosum and in the left inferior longitudinal and arcuate fasciculi positively predicted intelligence. Navas-Sánchez et al. (2014) also reported a positive correlation between fractional anisotropy (FA) values and intellectual capabilities in the corpus callosum. Overall, these findings demonstrate that intra-hemispheric long tracts connecting fronto-occipital and temporo-parietal regions as well as inter-hemispheric fibers are crucial for the integration of information.

The objective of this study was to identify and characterize WM microstructural differences between two HIQ children profiles in comparison with age-matched control subjects. To this end, tract-based spatial statistic (TBSS) was used to perform automated analysis of WM integrity (Smith et al., 2006). FA constitutes a sensitive marker of microstructural changes. Mean diffusivity (MD) as well as the axial (AD) and radial (RD) diffusivities (Tournier et al., 2011), allow a better interpretation of potential changes in terms of axonal integrity and myelination (Song et al., 2002).

We first hypothesized that HIQ children would show greater global WM integrity related to their general intelligence, when compared to control subjects. Second, the comparison of two profiles of HIQ children, namely Hom-HIQ, and Het-HIQ based on their PRI differences, would show local WM changes, potentially linked to their verbal and/or visuo-spatial abilities.

## Materials and methods

### Subjects

Participants in this study were children followed either at the children psychiatry department of Lyon's Neurological Hospital or the PSYRENE Center, both specialized in HIQ evaluation and psychological follow-up. Children were also recruited by placing ads in public schools (mostly for the recruitment of control subjects). Prior to enrollment, all children received a medical examination and were fully informed along with their parents of the study details. Children with any neurological, psychiatric disorders, learning disabilities such as dyslexia or dyspraxia, or MRI contra-indications were excluded from the study. None of the children received any medical treatments. Ethical committee approval (“CPP Sud-Est IV”) and written informed consent from the children and their parents were obtained. Three other subjects were excluded from this study: one for his inability to perform the MRI exam and two for motion artifacts.

All children underwent the WISC-IV with its four subscales [Verbal comprehension index (VCI), Perceptual reasoning index (PRI), Working memory index (WMI), and Processing speed index (PSI)] to calculate the FSIQ. Children scoring 130 or greater in VCI or PRI were included in HIQ groups. Children with a VCI significantly higher than the PRI were included in the Het-HIQ group, while those with no significant difference between VCI and PRI were included in the Hom-HIQ group. The significance of VCI-PRI differences was defined by the WISC-IV score with a range of 15–46 in the Het-HIQ group. Since the number of Het-HIQ children with significantly higher PRI than VCI was under represented (3 subjects out of 27), these subjects were excluded for group homogeneity purposes.

A total of 44 right-handed (according to the Edinburgh handedness scale Oldfield, 1971) HIQ children were included in this study. The study consisted of 8 girls and 36 boys aged between 8 and 12 years old [mean (± SD) age = 10.4 ± 1.3 y]. The Hom-HIQ group was composed of 20 children [5 girls and 15 boys; mean (±SD) age = 10.2 ± 1.2 y] with a FSIQ of 139.9 ± 11.1. The Het-HIQ group was composed of 24 children [3 girls and 21 boys, mean (±SD) age = 10.5 ± 1.4 y] with a FSIQ of 129.4 ± 10.6. Thirteen age-matched subjects [6 girls and 7 boys, mean (±SD) age = 10.5 ± 1.2 y] with a FSIQ of 105.2 ± 8.8 served as control subjects. Except for the two subjects excluded for motion artifacts, the quality of MRI data was optimal.

### Behavioral assessment

All children were assessed using the adapted French version of the Child Behavior Checklist (CBCL) for children from ages 4 to 16 (Achenbach, 1991). The CBCL is a 113-item parental report inventory that uses a Likert response format (not true, somewhat true, very true) for a variety of behaviors. The total Competence score was obtained from three subscales: activities, social, and school. The total Problem score was obtained from eight subscales: (I) withdrawn, (II) somatic complaints, (III) anxious, (IV) social, (V) thought, and (VI) attention problems, (VII) delinquent and (VIII) aggressive behavior. Two other subtotal scores, internal and external, were obtained from subscales (I+II+III) and (VII+VIII), respectively. Total scores were calculated by summing items, which was transformed by square root to approximate a normal distribution. Individual raw scores were converted into *T*-scores.

The Conners' Parent Rating Scale (CPRS) (Conners et al., 1998) was used to assess symptoms of Attention Deficit Hyperactivity Disorder (ADHD), and included six subscales: hyperactivity/impulsivity, psychosomatic, learning problems, anxiety, and conduct disorder. Individual raw scores were converted into *T*-scores.

### Image acquisition

MRI examinations were performed without any sedation at the MRI department of CERMEP-Imagerie du Vivant, on a 1.5T Siemens Sonata system (Erlangen, Germany) with an 8-channel head-coil and 40 mT/m gradients. The MRI protocol included a 3D T1-weighted magnetization prepared rapid gradient echo (MPRAGE) sequence (time of repetition/time of echo/time for inversion [TR/TE/TI] = 1,970/3.93/1,100 ms; flip angle = 15°; matrix size = 256 × 256; field of view = 256 × 256 mm; slice thickness = 1 mm; voxel size = 1 × 1 × 1 mm^3^; acquisition time = 8 min).

DTI protocol was based on a 2D multi-slice spin-echo echo-planar imaging (EPI) sequence (TR/TE = 6,900/86 ms, matrix size = 96 × 96, FOV = 240 × 240 mm, acquisition time = 7 min). Fifty-one contiguous axial slices, of 2.5 mm thickness were acquired in the anterior commissure—posterior commissure (AC-PC) plane. Twenty-four diffusion gradient directions (b = 1,000 s/mm^2^) were applied with a nominal isotropic resolution of 2.5 mm^3^. The b0 image was acquired four times to increase signal to noise ratio while the other directions were acquired twice. This protocol follows international recommendations on DTI acquisition (Jones, 2004).

Both T1-weighted, and DTI data were visually inspected (by S.H, D.S-M, and O.R) in order to detect artifacts arising from subject motion or scanner malfunction, and confirm the lack of visually detectable abnormalities.

### Data processing

Analysis of diffusion tensor data was performed using the Functional Magnetic Resonance Imaging of the Brain (FMRIB) Software Library (FSL) (Smith et al., 2004). Eddy current correction using FMRIB's Diffusion Toolbox (FDT) was first applied followed by a non-brain voxels extraction using the Brain Extraction Tool (BET) with a factor of 0.35. FA and MD as well as AD and RD maps were then generated. Individual DTI maps were visually inspected for the presence of significant residual motion or other artifacts.

TBSS analysis of FA maps first consisted in aligning all subjects' data into a common space by means of a nonlinear registration. Since children brain anatomy is slightly different from adult's, the common template used in FSL was thereby not adapted for our subjects. Therefore, all subjects' FA images were aligned to every other one in order to find the most “typical” subject as a target image to align all other. A mean FA image was then created and used to obtain a mean skeleton of the major WM tracts on which all aligned subjects FA data were then projected. The resulting data were next fed into a voxel-wise statistical analysis, performed to identify FA differences in areas between HIQ and Control subjects, and between Hom-HIQ and Het-HIQ groups, including age and sex as covariates. For a better assessment, TBSS was also applied to diffusion-derived data other than FA. The nonlinear warps and skeleton projection were therefore applied to AD, RD, and MD maps. The resulting warped maps were then merged and projected onto the original mean FA skeleton. Voxel-wise analysis was finally performed exactly as for the FA maps.

The employed voxel-wise analysis was based on a non-parametric approach using the permutation test theory with a standard generalized linear model design matrix. By allowing inference on the statistical maps when the null distribution is not known, this approach provides an easily implementable solution to the multiple testing problems. The permutation testing was performed using the Randomise module of FSL. Group comparisons were performed between each group of HIQ children and control subjects with age and gender as co-factors. The threshold-free cluster enhancement (TFCE) option of Randomise was applied on the resulting statistical maps. This method enhances cluster-like structures in an image without having to define an initial cluster-forming threshold or to carry out a large amount of data smoothing. The resulting statistical parameter maps were corrected for sex and age and for multiple comparisons by the family-wise error rate (FEW-corrected *p* < 0.05). The anatomical location of significant clusters was identified based on WM atlases (JHU ICBM-DTI81 White Mater Labels and JHU White-Matter Tractography Atlas) in FSL. In order to confirm the voxel-wise analysis results, quantitative values of diffusion metrics were extracted from 48 regions of interest (ROI) and 21 fiber-bundles of the JHU atlases, by multiplying atlas labels with the TBSS skeleton obtained from all 57 subjects.

Statistical analysis was performed using the R library (https://www.r-project.org). ANOVA followed by Tukey's *post-hoc* tests were performed on demographic data, IQ, CBCL, and CPRS scores, and DTI metrics (ROI and fiber bundles) to detect group-wise differences between the four children groups (Total-HIQ, Hom-HIQ, Het-HIQ, and Control) with age, gender and CBCL as co-factors.

## Results

Descriptive statistics of age and IQ scores are reported in Table 1. As groups were age-matched, there were no significant differences in age between any of the HIQ groups and the Control group. PRI values were significantly lower in Het-HIQ compared to Hom-HIQ groups, whereas no significant differences were observed for VCI values. As reported in Table 2, no significant differences were found in mean CBCL and CPRS T-scores when comparing HIQ and Control groups. However, some differences were found using an ANOVA test between mean Het-HIQ and Hom-HIQ groups in two CBCL subscales (I and V), namely the withdrawn (*p* = 0.07) and thought (*p* = 0.03) problems, as reported in Table 2.

**Table 1:** **Descriptive statistics (Mean ± ***SD***) in Control, heterogeneous-HIQ (Het-HIQ) and homogeneous-HIQ (Hom-HIQ) groups**.

	**Control**	**(*n* = 13)**	**Het-HIQ**	**(*n* = 24)**	**Hom-HIQ**	**(*n* = 20)**
Age	10.5 ± 1.2		10.5 ± 1.4		10.2 ± 1.2	
FSIQ	105.2 ± 8.8		129.4 ± 10.6	^***^	139.9 ± 11.1	^***^^###^
VCI	108.5 ± 6.9		144.5 ± 7.3	^**^	138.6 ± 11.4	^**^
PRI	99.8 ± 8.3		117.5 ± 12.0	^*^	136.4 ± 7.9	^*^^#^
WMI	96.1 ± 10.1		110.0 ± 14.0	^*^^##^	121.5 ± 16.2	^***^
PSI	104.0 ± 15.1		104.1 ± 13.8		114.6 ± 17.4	

* p < 0.05;

** p < 0.01;

*** p < 0.001 between HIQ and Control groups;

# p < 0.05;

## p < 0.01;

### *p < 0.001 between Hom-HIQ and Het-HIQ groups*.

**Table 2:** **Normalized T-scores (Mean ± ***SD***) measured in Competence and Problem subscales of the Child Behavior Checklist (CBCL) and in Conners' Parent Rating Scale (CPRS) questionnaires in Control, heterogeneous-HIQ (Het-HIQ) and homogeneous-HIQ (Hom-HIQ) groups**.

	**CBCL**					**CPRS**
	**Competence**	**Withdraw Pb**.	**Thought Pb**	**Attention Pb**.	**Problem**	**Total**
Control	37.0 ± 4.4	74.1 ± 16.1^*^	65.0 ± 8.3	64.7 ± 9.9	59.8 ± 9.0	58.0 ± 11.9
Het-HIQ	35.5 ± 5.2	76.3 ± 16.3^*^	71.0 ± 11.7^*^	72.2 ± 14.6^*^	62.4 ± 11.1	56.4 ± 9.2
Hom-HIQ	33.6 ± 6.1	64.4 ± 14.5	63.8 ± 8.5	65.4 ± 12.6	55.1 ± 11.3	51.0 ± 9.1

*Normalized T-scores of Total Competence tests are abnormal if < 30*.

* *Normalized T-scores of CBCL subscales are abnormal if > 70*.

### Voxel-wise analysis

**Figure 1:**
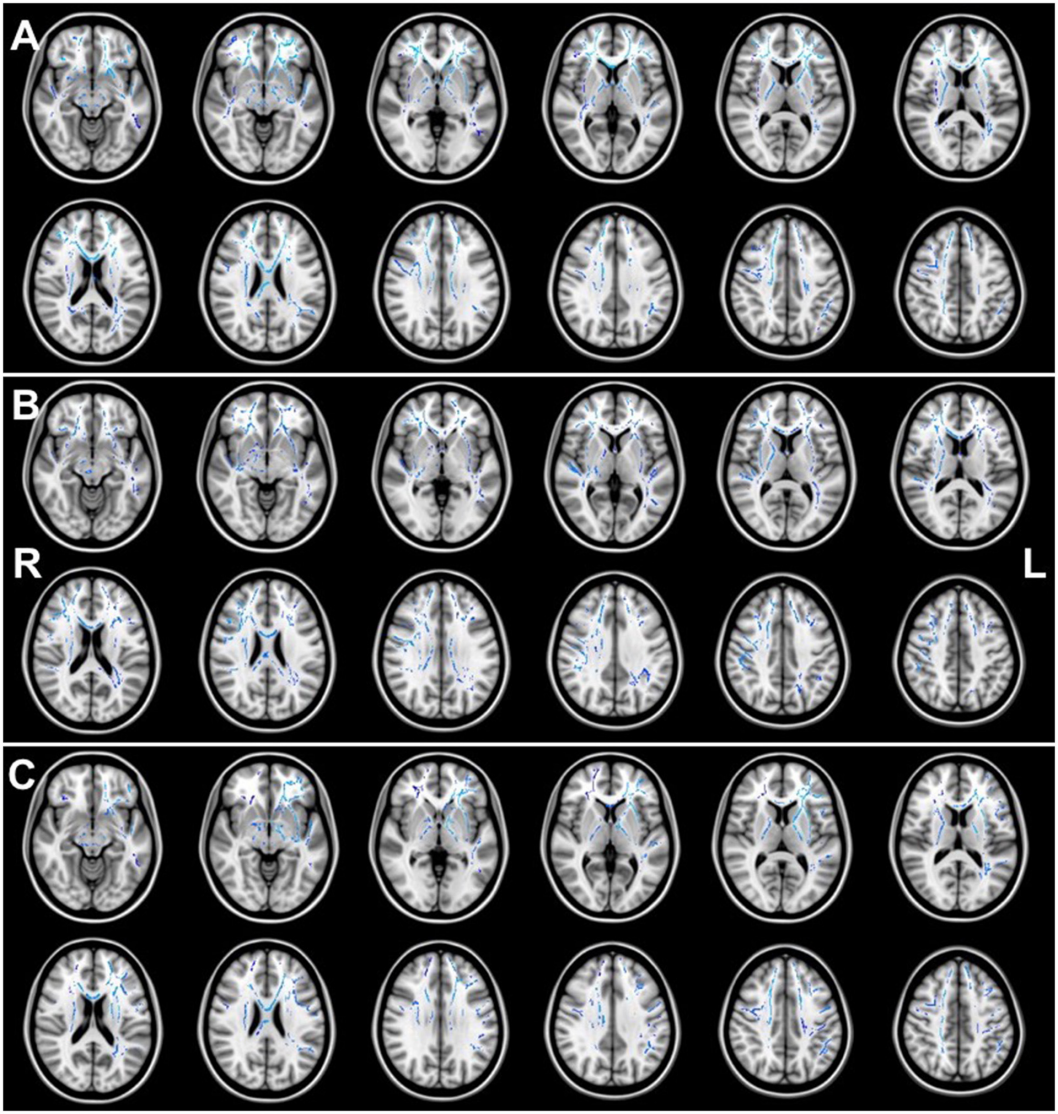
**Regions of significant (***p*** < 0.05) greater AD (blue) when comparing (A)** the Total-HIQ group (Tot-HIQ), **(B)** the homogeneous-HIQ (Hom-HIQ) group and **(C)** the heterogeneous-HIQ (Het-HIQ) group with the Control group.

**Figure 2:**
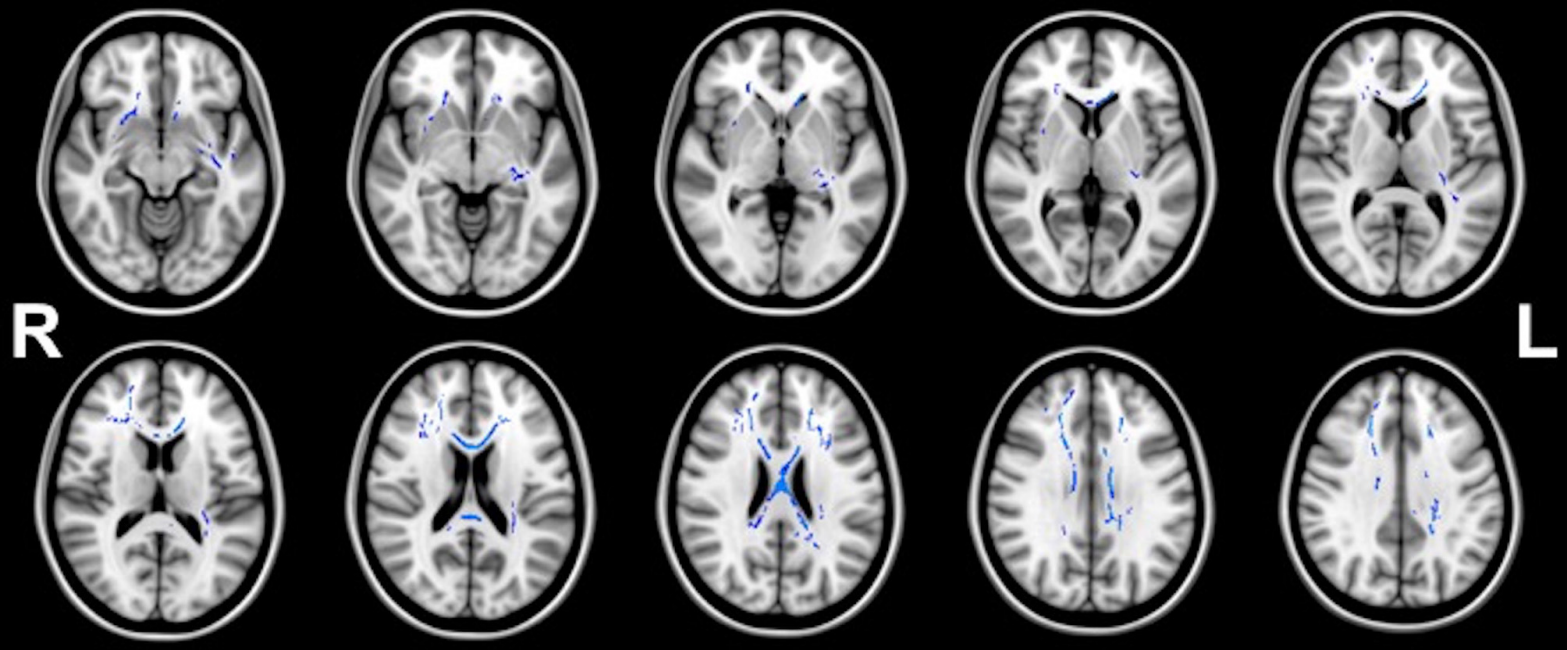
**Regions (blue) of significant (***p*** < 0.05) greater FA in homogeneous-HIQ (Hom-HIQ) compared to heterogeneous-HIQ (Het-HIQ) children**.

Figures 1, 2 illustrate the significant AD and FA differences observed when comparing HIQ and Control groups. No significant differences were reported for MD and RD diffusion metrics in any comparison.

#### Axial diffusivity (AD)

When compared to the Control group, TBSS analysis showed significant higher AD values in Total-HIQ, Hom-HIQ, and Het-HIQ groups as illustrated in Figure 1. For quantification purposes, the percentage of significant voxels was calculated in each ROI of the ICBM-DTI81 JHU atlas (Table 3). The three groups showed a large number of significant voxels (above 50% of ROI volume) in many WM regions, including mainly the central structures such as the fornix and the corpus callosum as well as the brainstem (cerebral peduncles and medial lemnisci) and cerebellar structures (cerebellar peduncles). In the corpus callosum, a decreasing gradient was observed from genu to splenium that was slightly more pronounced in the Het-HIQ group. The larger changes included different regions of the corona radiata and the capsule as well as different parts of WM fasciculi, namely the uncinate (UNC), the superior fronto-occipital (SFO), the posterior thalamic radiations (PTR), the cortico-spinal tract (CST) and the superior longitudinal (SLF) (Table 3).

**Table 3:** **Number of significant voxels (N_**sign**_) and percentage (%) of significant voxels per total number of voxels (N_**total**_) per ROI of the JHU atlas (ICBM-DTI81 White Matter Labels) with significant changes in AD values when comparing Total-HIQ (Tot-HIQ), homogeneous-HIQ (Hom-HIQ) and heterogeneus-HIQ (Het-HIQ) groups with the Control (C) group, and in FA values when comparing Hom-HIQ and Het-HIQ groups**.

		**AD Tot-HIQ>C**			**AD Hom-HIQ>C**			**AD Het-HIQ>C**			**FA Hom-HIQ>Het-HIQ**		
**ROIs**	**L/R**	**N_total_**	**N_sign_**	**%**	**N_total_**	**N_sign_**	**%**	**N_total_**	**N_sign_**	**%**	**N_total_**	**N_sign_**	**%**
GCC		1,630	1,138	70	1,528	817	53	1,443	687	48	1,495	634	42
BCC		2,777	1,781	64	2,606	1,458	56	2,608	1,576	60	2,970	1,813	61
SCC		1,069	273	26	878	340	39	916	68	7	1,500	574	38
CP	R	543	354	65	446	278	62	484	238	49	310	0	0
	L	387	267	69	335	0	0	392	278	71	238	0	0
SCP	R	148	115	78	46	11	24	132	108	82	84	0	0
	L	189	95	50	88	31	35	168	74	44	128	0	0
ICP	R	46	12	26	44	0	0	17	11	65	135	0	0
	L	109	26	24	111	49	44	82	0	0	123	0	0
SCR	R	1,300	846	65	1,116	531	48	1,312	779	59	1,199	49	4
	L	972	335	34	654	35	5	1,229	442	36	796	26	3
ACR	R	1,437	947	66	1,374	834	61	1,313	446	34	1,412	492	35
	L	1,558	1,178	76	1,590	1,018	64	1,610	1,175	73	1,138	236	21
PCR	R	353	165	47	584	164	28	272	55	20	563	0	0
	L	450	164	36	757	380	50	452	79	17	627	253	40
ALIC	R	611	273	45	599	280	47	632	263	42	492	0	0
	L	731	454	62	649	61	9	732	469	64	512	0	0
PLIC	R	791	587	74	704	446	63	762	528	69	464	0	0
	L	724	353	49	466	5	1	810	513	63	303	0	0
RLIC	R	530	222	42	661	327	49	301	6	2	558	0	0
	L	400	147	37	550	212	39	398	56	14	619	219	35
EC	R	900	292	32	860	484	56	756	7	1	1,177	231	20
	L	1,270	764	60	1,281	784	61	1,281	773	60	975	19	2
ML	R	101	61	60	108	34	31	121	80	66	34	0	0
	L	102	62	61	124	50	40	121	75	62	28	0	0
TT	R	118	67	57	21	0	0	101	0	0	92	0	0
	L	10	6	60	0	0	0	12	6	50	16	2	13
UNC	R	34	0	0	26	1	4	20	0	0	67	0	0
	L	70	36	51	70	41	59	69	17	25	51	26	51
SFOF	R	32	6	19	11	2	18	42	28	67	92	0	0
	L	46	26	57	79	2	3	48	32	67	51	0	0
FX		161	74	46	119	47	39	52	7	13	165	0	0
FX/ST	R	55	4	7	36	10	28	1	0	0	128	0	0
	L	58	11	19	77	6	8	45	7	16	307	150	49
PTR	R	615	108	18	555	71	13	490	0	0	667	0	0
	L	884	401	45	504	339	67	852	294	35	631	16	0
CST	R	182	24	13	152	23	15	136	9	7	43	0	0
	L	211	96	45	126	0	0	191	17	9	53	0	0
SLF	R	651	81	12	862	427	50	443	32	7	1,268	0	0
	L	547	89	16	778	84	11	700	228	33	1,426	0	0
CCG	R	18	1	6	17	0	0	19	2	11	92	9	10
	L	14	0	0	47	0	0	9	0	0	184	85	46

*L, Left; R, Right; MCP, Middle cerebellar peduncle; GCC, Genu of corpus callosum; BCC, Body of corpus callosum; SCC, Splenium of corpus callosum; FX, Fornix (column and body of fornix); CST, Corticospinal tract; CP, Cerebral peduncle (Brainstem); ML, Medial lemniscus (Brainstem); ICP, Inferior cerebellar peduncle; SCP, Superior cerebellar peduncle; ALIC, Anterior limb of internal capsule; PLIC, Posterior limb of internal capsule; RLIC, Retrolenticular part of internal capsule; EC, External capsule; ACR, Anterior corona radiata; SCR, Superior corona radiata; PCR, Posterior corona radiata; PTR, Posterior thalamic radiation; SS, Sagittal stratum; CCG, Cingulum cingulate gyrus; FX/ST, Fornix (cres)/Stria terminalis; SLF, Superior longitudinal fasciculus; SFOF, Superior fronto-occipital fasciculus; IFOF, Inferior fronto- occipital fasciculus; UNC, Uncinate fasciculus; TT, Tapetum*.

When comparing the localization of these AD increases, we observed a significant difference in hemispheric lateralization. As illustrated in Figure 1, 56% of significant voxels were found in the right hemisphere of Hom-HIQ group while 58% were found in the left hemisphere of Het-HIQ, leading to a left/right ratio of 0.78 and 1.38 in Hom-HIQ and Het-HIQ groups, respectively.

#### Fractional anisotropy (FA)

TBSS analysis showed significant higher FA values in Hom-HIQ compared to Het-HIQ groups (Figure 2). FA differences were observed mainly in the corpus callosum and several central regions such as the anterior and posterior parts of the corona radiata (CR), and the internal and external capsules. Further, several parts of WM fasciculi such as the left uncinate (UNC), the left fornix stria terminalis (FX/ST) and the cingulum cingulate gyrus (CCG) were significantly detected. No significant decreases of FA were observed.

### Quantitative analysis

Based on the JHU atlases, the diffusion metrics were measured in different ROIs (ICBM DTI-81) and fiber-bundles (White-Matter Tractography) in HIQ and Control groups to confirm our voxel-wise findings. When comparing Hom-HIQ and Het-HIQ groups, FA values were significantly increased in several ROIs, the body of corpus callosum, the left inferior cerebellar peduncle (ICP), the left posterior corona radiata (PCR), and the right fornix stria terminalis (FX/ST), and in one fiber bundle, the cingulum cingulate gyrus (CCG). When comparing with the Control group, AD values were significantly increased in 17 ROIs and six fiber bundles of the Tot-HIQ group, in four ROIs and three fiber bundles of the Hom-HIQ group, and four ROIs and two fiber bundles of the Het-HIQ group, as reported in Table 4.

**Table 4:** **Axial diffusivity (AD) values (Mean ± ***SD***) in selected ROIs (ICBM-DTI81 White Matter Labels) and fiber-bundles (White-Matter Tractography) of JHU atlases applied to Control, heterogeneous-HIQ (Het-HIQ), homogeneous-HIQ (Hom-HIQ), and Total-HIQ (Tot-HIQ) groups**.

**ROIs**	**Tot-HIQ**	**Hom-HIQ**	**Het-HIQ**	**Control**
BCC	1.468 ± 0.050^**^	1.475 ± 0.058^**^	1.463 ± 0.042^*^	1.422 ± 0.043
SCP	1.431 ± 0.051^*^	1.444 ± 0.055	1.419 ± 0.044	1.403 ± 0.038
L-ICP	1.099 ± 0.038^**^	1.115 ± 0.035^***^^#^	1.086 ± 0.035	1.064 ± 0.030
L-SCP	1.278 ± 0.057^*^	1.286 ± 0.0523	1.272 ± 0.060	1.245 ± 0.033
R-CP	1.306 ± 0.050^*^	1.311 ± 0.047	1.302 ± 0.053	1.273 ± 0.038
L-CP	1.361 ± 0.044^*^	1.356 ± 0.040	1.365 ± 0.048^*^	1.322 ± 0.056
R-ALIC	1.158 ± 0.047^*^	1.164 ± 0.045	1.154 ± 0.050	1.129 ± 0.039
L-ALIC	1.163 ± 0.048^*^	1.160 ± 0.048	1.165 ± 0.050^*^	1.125 ± 0.043
R-PLIC	1.255 ± 0.042^*^	1.256 ± 0.047	1.255 ± 0.039	1.227 ± 0.042
L-RLIC	1.297 ± 0.042^*^	1.305 ± 0.037^*^	1.290 ± 0.046	1.268 ± 0.045
R-ACR	1.151 ± 0.039^*^	1.159 ± 0.037^*^	1.144 ± 0.040	1.121 ± 0.042
L-ACR	1.154 ± 0.039^*^	1.157 ± 0.030^*^	1.153 ± 0.045	1.121 ± 0.044
L-SCR	1.188 ± 0.062	1.181 ± 0.054	1.194 ± 0.069	1.155 ± 0.050
L-PCR	1.233 ± 0.043^*^	1.240 ± 0.038^*^	1.227 ± 0.047	1.199 ± 0.043
L-PTR	1.338 ± 0.052^**^	1.338 ± 0.049^*^	1.338 ± 0.055^*^	1.293 ± 0.040
L-SS	1.256 ± 0.053^*^	1.259 ± 0.044	1.254 ± 0.061	1.216 ± 0.055
L-EC	1.181 ± 0.039^*^	1.183 ± 0.036	1.800 ± 0.042	1.146 ± 0.040
R-FX/ST	1.165 ± 0.052	1.182 ± 0.058	1.151 ± 0.043	1.152 ± 0.077
L-SLF	1.169 ± 0.042^**^	1.167 ± 0.036	1.170 ± 0.047	1.139 ± 0.030
**Fiber bundles**	**Tot-HIQ**	**Hom-HIQ**	**Het-HIQ**	**Control**
L-CCG	1.137 ± 0.039	1.142 ± 0.035	1.132 ± 0.042	1.112 ± 0.041
L-CST	1.236 ± 0.032^*^	1.231 ± 0.029	1.240 ± 0.034^*^	1.211 ± 0.030
Fmin (GCC)	1.236 ± 0.040^*^	1.245 ± 0.039^*^	1.229 ± 0.039	1.206 ± 0.039
L-IFOF	1.197 ± 0.033^*^	1.198 ± 0.028	1.196 ± 0.037	1.179 ± 0.017
L-ILF	1.219 ± 0.036^**^	1.219 ± 0.035	1.219 ± 0.037	1.196 ± 0.023
L-SLF	1.137 ± 0.035^*^	1.133 ± 0.031	1.140 ± 0.039	1.116 ± 0.027
L-UNC	1.170 ± 0.033^**^	1.173 ± 0.028^**^	1.167 ± 0.035^*^	1.136 ± 0.034
R-UNC	1.161 ± 0.032	1.174 ± 0.030^*^^#^	1.149 ± 0.029	1.142 ± 0.042

*Values in bold are significant*.

* P < 0.05;

** p < 0.01;

*** p < 0.001 between HIQ and Control groups;

# *p < 0.05; between Het-HIQ and Hom-HIQ groups*.

*L, Left; R, Right; ROIs: BCC, Body Corpus Callosum; GCC, Genu Corpus Callosum; SCC, Splenium Corpus Callosum; ICP, Inferior cerebral Peduncle; CP, Cerebellum Peduncle; ACR, Anterior Corona radiate; PCR, Posterior Corona radiate; PTR, Posterior Thalamic Radiations; SS, Striatum Sulcus; EC, External Capsule; SLF, Superior Longitudinal Fasciculus; Fiber-bundles: CCG, Cingulum Cingulate Gyrus; CST, Left Cortico-Spinal Tract; Fmin, Minor Forceps; Unc, Uncinate fasciculus*.

Effects of full-scale IQ, and CBCL score (Achenbach) on AD values were estimated using General linear models for WM region reported in Table 4. The relative importance of these predictors was estimated for each WM region. A mean value of 77.88% (78.21% for JHU and 77.74% for ROI) and 22.12% (21.79% for JHU and 22.26% for ROI) was obtained for the full-scale IQ and CBCL score respectively. These results showed that differences observed in WM between HIQ and Control groups are mainly driven by IQ.

## Discussion

In this work, TBSS analysis was performed to investigate the diffusion-related differences in HIQ children, and between two groups of HIQ, based on their IQ profile, namely homogeneous and heterogeneous. To our knowledge, this work is the first to investigate WM microarchitecture in these two profiles of HIQ children.

### Global changes with intelligence

When compared to the Control group, both HIQ groups presented greater AD and FA in widespread WM regions of frontal, central and associative pathways. Central regions included mainly the body and the genu of the corpus callosum, the fornix and the cingulum as well as brainstem and cerebellar regions. These findings are in agreement with previous DTI studies on intelligence showing a bilateral fronto-parietal network as a neural substrate of enhanced information processing and intelligence (Dunst et al., 2014; Navas-Sánchez et al., 2014). Similarly, a previous fMRI study demonstrated the use of more extensive and bilateral cortical connections in gifted adolescents (Desco et al., 2011). Such improvement of bilateral and frontal WM organization contributes to improved cognitive performance in working memory, fluency, and executive functioning such as inhibition and task-switching (Seghete et al., 2013). Other studies suggested that corpus callosum related changes are crucial for higher cognitive capabilities (Singh and O'Boyle, 2004; Hutchinson et al., 2009; Prescott et al., 2010). Also, it has been demonstrated that the genu (Sidtis et al., 1981) and the splenium (Funnell et al., 2000) are involved in high order transfer of semantic information. In addition, the higher FA in the fornix may reflect a greater episodic memory (Gaffan and Wilson, 2008). Projection fibers of the capsule and the corona radiata also showed higher FA. These fibers regroup ascending fibers from the thalamus and descending fibers from the fronto-parietal cortex to subcortical nuclei. They constitute the backbone of perceptual and motor functions and other cognitive functions (Schmahmann and Pandya, 2008). Additionally, our study showed greater FA and AD in other regions of associative WM fasciculi, namely the uncinate, the superior longitudinal (SLF) and the superior fronto-occipital (SFOF). The SLF supports bidirectional networks involved in high order cognitive executive functions such as attention, inhibition as well as memory and language (Mesulam, 1998) while the uncinate (UNC) fasciculus may play a prominent role in semantic tasks (Catani and Mesulam, 2008; Catani et al., 2013). Thus, these findings show that both inter- and intra-hemispheric connections are enhanced in HIQ children suggesting that they may benefit from a better WM integrity, providing higher performances in most abilities.

### Local changes with HIQ profiles

When comparing Hom-HIQ and Het-HIQ groups to the Control group, we found AD increases in numerous WM regions. However, their spatial distribution was clearly different between the two HIQ groups. The Het-HIQ group showed more AD changes in the left hemisphere in contrast to the Hom-HIQ group which showed more AD differences in the right hemisphere.

The left hemisphere is well known to play a major role in lingual functions (Szaflarski et al., 2012) with a strong involvement of the SLF, the SFOF, and UNC fasciculi (Catani et al., 2002). In Het-HIQ group, AD is only significantly increased in the left UNC fasciculus while in Hom-HIQ group, it is also increased the right UNC, which is involved in emotion processing and memory, and plays an important role in lexical retrieval, semantic associations and naming aspects of the language (Grossman and Ash, 2004; Agosta et al., 2010; Galantucci et al., 2011). Thus, the left lateralization of increased diffusivity observed in Het-HIQ children can be associated to their high language performances (high VCI) in contrast to their relatively low visuo-spatial capacities (low PRI). Indeed, the right hemisphere is more specialized in visuo-spatial capacities such as facial recognition and attention monitoring (Gazzaniga, 2000; de Schotten et al., 2011; Caeyenberghs and Leemans, 2014).

Recently, Marinsek et al. (2014) proposed that both hemispheres diverge by their reasoning strategies. On one hand, the left hemisphere is considered as an interpreter, tending to reduce uncertainty by creating explanations, filling gaps of information, and making inference (Braun and Suffren, 2011). On the other hand, the right hemisphere is considered as a realist, striving to reduce inconsistencies between hypotheses and reality by detecting conflicts, updating information, supporting mental set shifting (Goel et al., 2013), and monitoring and inhibiting thoughts and behavior (Chatham et al., 2012). Moreover, Vendetti et al. (2015) found hemispheric differences in relational reasoning. Indeed, the left hemisphere has been shown to excel in relational encoding, particularly when the information is ordered linearly. It interacts more with itself, whereas the right hemisphere demonstrates connectivity patterns associated with both hemispheres (Gotts et al., 2013), and acts more as a monitor of evaluation and inhibition.

However, the complementarity or independence of hemispheric specialization remains an open question (Badzakova-Trajkov et al., 2016). The causal hypothesis suggests that strengths in language and weaknesses in visuo-spatial abilities of Het-HIQ may result from an earlier development of language, which thereby reinforces a hemispheric asymmetry specialization. Also, disabilities observed in Het-HIQ children may derive from an initial sub-development of the right hemisphere due to genetic and/or environmental causes.

### Methodological consideration

FA is a useful measure of brain WM integrity that can be derived from DTI. It provides a simple and robust mean to assess the degree of anisotropic diffusion occurring within a region. FA is determined by both microscopic factors, such as myelination and axonal density, and macroscopic factors such as crossing fibers. Thus, the greater values of FA in HIQ children could be interpreted in terms of higher directionally coherent organization of fibers within voxels (Beaulieu, 2002), increased fiber tracts myelination (Jones et al., 2013), or higher axonal density and/or diameter (Mori and Zhang, 2006). However, increased FA could also be associated with less integrity, due to neurodegenerative processes in crossing fibers' regions (Douaud et al., 2011). Nevertheless, this effect is unlikely to occur in children. Furthermore, the increased FA observed in HIQ children is supported by the higher AD found in all HIQ groups compared to controls. AD quantifies the amount of water diffusion along the primary axis of the axon. It is believed to better reflect axonal density and/or caliber, while RD constitutes a more specific marker of myelin integrity (Song et al., 2005; Jones et al., 2013). Based on these assumptions, our findings suggest that HIQ children might present a greater axonal density or caliber than control subjects. The higher degree of axonal density or caliber could increase the signal transmission speed and thereby might account for more efficient brain functioning.

Among the diffusion metrics, significant differences were observed in FA and AD, but not in MD and RD. We can hypothesize that RD values might be decreased in HIQ children, leading to insignificant changes in MD but significant increase in FA. Such a hypothetical RD decrease would reinforce the argument of a higher degree of myelination in relation with intelligence (Dunst et al., 2014).

Nevertheless, this study may suffer from the relatively small number of children in each group. This is mostly related to the targeted population of children, which is difficult to recruit. Also, the ratio of girls and boys was different between HIQ and Control groups. This bias is probably related to girls' observed ability of “over-adaptation” which leads to less clinical consultations, thus reducing the number of girls diagnosed as HIQ, and particularly as Het-HIQ. In contrast, our control group was well balanced in that matter. This potential bias was taken into account by including age and sex as covariates in the voxel-wise statistical analysis.

## Conclusion

Our findings demonstrate that increased WM integrity is correlated with higher intelligence capabilities, particularly in main central structures such as the corpus callosum, but also in WM associative and projection pathways. Our results highlight significant differences in WM tissue integrity between HIQ profiles, mainly in terms of hemispheric lateralization. Regions of higher diffusivity were more distributed in the right hemisphere of HIQ children with homogeneous IQ and in the left hemisphere of HIQ children with heterogeneous IQ. These findings suggest a potential relationship between intelligence profile and lateralization. The left hemispheric specialization of Het-HIQ children suggests a better capacity in language and may reflect their independent and self-connected behavior. In contrast, the bilateral and right hemispheric organization of Hom-HIQ children can support their greater capacities in exploration, adaptation and learning from environmental stimulations.

## Ethics statement

This study was carried out in accordance with the recommendations of CPP Sud-Est IV (France) with written informed consent from all subjects. All subjects gave written informed consent in accordance with the Declaration of Helsinki. The protocol was approved by the CPP Sud-Est IV (France).

## Author contributions

FN coordinated the organization and implementation of the study, recruited the children, provided the clinical expertise in the interpretation of the results, and participated to the paper writing. SH performed the voxelwise analysis and participated to the statistical analysis, and to the paper writing. GK performed the regional analysis and participated to the statistical analysis, and to the paper writing. CS helped in the interpretation of the results and participated to the paper writing. PF participated to the coordination of the study. OR participated to the coordination of the study, recruited children and provided the clinical expertise in the interpretation of the results. DSM supervised MR acquisition, provided methodological expertise in the interpretation of the results, and participated to the paper writing.

### Conflict of interest statement

The authors declare that the research was conducted in the absence of any commercial or financial relationships that could be construed as a potential conflict of interest.

## Abbreviations

AD: axial diffusivity
ADHD: attention deficit hyperactivity disorder
CBCL: child behavior checklist
CPRS: Conners' parent rating scale
DTI: diffusion tensor imaging
FSIQ: full scale intelligence quotient
Het-HIQ: heterogeneous-high intelligence quotient
HIQ: high intelligence quotient
Hom-HIQ: homogeneous-high intelligence quotient
MD: mean diffusivity
PRI: perceptual reasoning index
PSI: processing speed index
RD: radial diffusivity
TBSS: tract-based spatial statistic
TE: time of echo
TI, time for inversion. TR: time of repetition
VCI: verbal comprehension index
WISC: Wechsler intelligence scale for children
WM: white matter
FA: fractional anisotropy
WMI: working memory index.
